# The B-type Channel Is a Major Route for Iron Entry into the Ferroxidase Center and Central Cavity of Bacterioferritin*

**DOI:** 10.1074/jbc.M114.623082

**Published:** 2014-12-15

**Authors:** Steve G. Wong, Jason C. Grigg, Nick E. Le Brun, Geoffrey R. Moore, Michael E. P. Murphy, A. Grant Mauk

**Affiliations:** From the ‡ Department of Biochemistry and Molecular Biology, the Centre for Blood Research and; § Department of Microbiology and Immunology, University of British Columbia, Vancouver, British Columbia V6T 1Z3, Canada and; ¶ Centre for Molecular and Structural Biochemistry, School of Chemistry, University of East Anglia, Norwich NR4 7TJ, United Kingdom

**Keywords:** Escherichia coli (E. coli), Ferritin, Iron, Site-directed Mutagenesis, X-ray Crystallography, Bacterioferritin, Iron Storage, Iron Core

## Abstract

Bacterioferritin is a bacterial iron storage and detoxification protein that is capable of forming a ferric oxyhydroxide mineral core within its central cavity. To do this, iron must traverse the bacterioferritin protein shell, which is expected to occur through one or more of the channels through the shell identified by structural studies. The size and negative electrostatic potential of the 24 B-type channels suggest that they could provide a route for iron into bacterioferritin. Residues at the B-type channel (Asn-34, Glu-66, Asp-132, and Asp-139) of *E. coli* bacterioferritin were substituted to determine if they are important for iron core formation. A significant decrease in the rates of initial oxidation of Fe(II) at the ferroxidase center and subsequent iron mineralization was observed for the D132F variant. The crystal structure of this variant shows that substitution of residue 132 with phenylalanine caused a steric blockage of the B-type channel and no other material structural perturbation. We conclude that the B-type channel is a major route for iron entry into both the ferroxidase center and the iron storage cavity of bacterioferritin.

## Teaser

**Background:** Routes of iron entry into prokaryote ferritins are unknown.

**Results:** Mutation of the B-type channel of *E. coli* bacterioferritin resulted in substantially decreased rates of Fe(II) oxidation and mineralization.

**Conclusion:** The B-type channel is an important route for iron entry into bacterioferritin.

**Significance:** B-type channels may be of general importance as routes of iron uptake in prokaryotic ferritins.

## Introduction

Bacterioferritin (BFR)^2^ is a bacterial member of the ferritin family that is composed of 24 identical subunits and that is distinguished from the other members of this family by its ability to bind protoheme IX at 12 intersubunit sites. The subunits of BFR oligomerize to form a spherical structure (outer diameter ∼120 Å) with a hollow, central cavity (inner diameter ∼80 Å) such that the Met-52 residues of adjacent pairs of monomers coordinate the heme iron by means of bismethionine axial ligation. Each subunit of BFR possesses a dinuclear iron site (the ferroxidase center) that catalyzes the oxidation of Fe^2+^ to Fe^3+^ by dioxygen to endow the protein with its characteristic ferroxidase activity. This activity promotes the formation of the insoluble “iron core” that occupies the central cavity of the protein and that can accommodate a maximum of ∼2700 atoms of iron per 24-mer (1). These functional properties of BFR enable it to serve the dual function of iron storage and iron detoxification.

Facilitation of iron core formation by the ferroxidase activity of BFR occurs by means of three kinetic phases (2, 3). Phases 1 and 2 involve binding and subsequent oxidation of Fe^2+^ at the dinuclear iron sites located at the center of each subunit. The third and slowest phase involves the formation of the iron core, a process that is initiated by binding of iron to the inner surface of the protein at a location adjacent to the dinuclear site (nucleation) (4, 5) followed by growth of the iron core. Significantly, occupation of the dinuclear iron site by iron is critical for core formation in *Escherichia coli* BFR.

**FIGURE 1.**
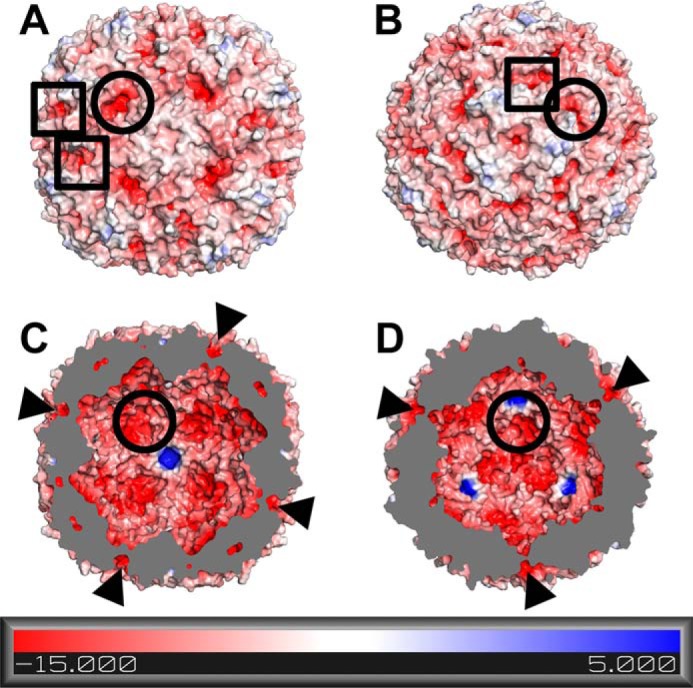
**Electrostatic potential surfaces of *E. coli* bacterioferritin.** The electrostatic potential surface diagrams of the outer surface (*A* and *B*) and inner surface (*C* and *D*) of BFR (PDB ID 3E1J (4)) calculated by APBS are shown. The protein is oriented so that the 4-fold channel (*A* and *C*) or 3-fold channel (*B* and *D*) is in the center. Locations of representative B-type channels and ferroxidase pores are identified with *circles* and *squares*, respectively. B-type channels in the cross sectional views (*C* and *D*) are also indicated with *arrows*.

To form the iron core, iron must first traverse the protein shell through one or more currently unidentified routes. The three-dimensional structure of BFR reveals the occurrence of eight 3-fold symmetry channels, six 4-fold symmetry channels, and 24 B-type channels (6, 7), all of which are potential routes of iron entry (Fig. 1). The 3-fold symmetry channels of *E. coli* BFR are lined with the charged residues Asp-109, Arg-117, and Asp-118, the 4-fold symmetry channels are lined with the polar residues Asn-148 and Gln-151, and the B-type channels are lined with the charged or hydrophilic residues Asp-132, Glu-135, Thr-136, and Asp-139 from one subunit and with Asn-34 and Glu-66 from adjacent subunits. So-called ferroxidase pores (6, 7) are also present that connect bulk solvent outside the protein shell with the dinuclear iron sites. These pores are significantly larger than the other channels and are lined with residues that are primarily hydrophobic in nature: Asn-17, Val-20, Ala-21, Leu-93, Asp-96, and Gly-97.

In comparison, the human H-chain ferritin 3-fold symmetry channels are lined with negatively charged residues (Asp-131 and Glu-134), the 4-fold symmetry channels are lined with largely hydrophobic residues (Leu-165, Leu-169, and His-173), and the ferroxidase pores are lined primarily with hydrophobic residues (Leu-26, Tyr-29, Ala-30, Leu-106, and Val-110). Interestingly, the B-type channels occur in the structures of prokaryotic ferritins (6–8) and ferritin from one diatom (9), but such channels are not present in other eukaryotic ferritins. The 3-fold symmetry channels of mammalian ferritins are believed to be the primary routes of iron entry into and exit from the iron core of these proteins (10–13). Substitution of Asp-131 and Glu-134 in human H-chain ferritin resulted in decreased iron oxidation activity and iron binding at the dinuclear iron sites as determined by isothermal titration calorimetry and fluorescence quenching experiments (10–12). Electrostatic potential calculations also support the role of the 3-fold channels in mammalian ferritins as an iron entry route (13).

The primary routes of iron entry into and exit from the iron storage cavity of prokaryotic ferritins remain unidentified. In *E. coli* BFR, the similarity of the 3-fold symmetry channels to the corresponding channels of mammalian ferritins make them attractive candidates for this role, but previous attempts to disrupt these channels in BFR were inconclusive (14). Support for the 4-fold symmetry channels as iron entry routes is provided by the crystal structures of *Azotobacter vinelandii* BFR in which barium and iron are found to bind at the 4-fold symmetry channel (15). However, electrostatic potential calculations with *E. coli* BFR show the 4-fold symmetry channels to be positively charged when viewed from the inner surface of the protein (Fig. 1). On the other hand, calculations of channel sizes and electrostatic potentials suggest that the B-type channels of BFRs exhibit structural characteristics that should facilitate the transfer of iron from bulk solvent into the interior of BFR (Fig. 1). The ability of the B-type channels to attract metal cations is demonstrated by the crystal structures of BFRs from *A. vinelandii* and *Mycobacterium smegmatis*, both of which bind Mg^2+^ at the B-type channels (15, 16).

From the analysis provided above, the B-type channels appear to be a likely route for iron transfer from bulk solvent into the inner cavity of BFR so that disruption of these channels should lead to a decrease in iron mineralization rates (Phase 3 of BFR ferroxidase activity). To evaluate this possibility, residues in these channels have been substituted either to reduce negative charge, introduce steric hindrance, or both, and the effects of these substitutions on the kinetics of BFR ferroxidase activity have been evaluated.

## EXPERIMENTAL PROCEDURES

### 

#### 

##### Bacterial Strains, Site-directed Mutagenesis, and Protein Purification

*E. coli* strain AL1 (BL21(DE3) *bfr*^−^) (1) transformed with pALN1 (pET21a containing *E. coli bfr* gene (4)) was used for expression of wild-type *E. coli* BFR. Site-directed mutagenesis reactions were performed with a QuikChange mutagenesis kit (Stratagene) to produce the N34F, E66F, D132F, D132N, and D139F BFR variants, and the mutations were verified by DNA sequence analysis (Genewiz).

Starter cultures were grown overnight in LB media containing ampicillin (0.1 mg/ml) and kanamycin (0.05 mg/ml). Erlenmeyer flasks (2 liters) containing LB media (1 liter) and ampicillin (0.1 mg/ml) were inoculated with the starter cultures and grown at 37 °C with 250 rpm shaking. Expression of BFR was induced with the addition of isopropyl 1-thio-β-d-galactopyranoside (1 mm) when the culture *A*_600_ was ∼1. Cells were harvested by centrifugation 10–15 h after induction, and the cell pellet was washed with and resuspended in potassium phosphate buffer (50 mm, pH 7.2). The cells were lysed with an Avestin Emulsiflex-C5 high pressure homogenizer. The lysed samples were incubated at 65 °C for 15 min and placed on ice before centrifugation to remove cell debris and denatured proteins. The supernatant fluid was applied to an anion exchange column (Q-Sepharose Fast Flow (GE Healthcare)) that had been equilibrated with potassium phosphate buffer (50 mm, pH 7.2) and that was subsequently developed with an ÄKTA Purifier system (GE Healthcare). The column was washed with potassium phosphate buffer (50 mm, pH 7.2), and the protein was eluted with a linear gradient of NaCl (0–0.5 m). Fractions containing BFR were pooled, concentrated, and loaded onto a gel filtration column (HiLoad Superdex-200 Preparation Grade (GE Healthcare)) that had been equilibrated with potassium phosphate buffer (50 mm, pH 7.2) containing NaCl (0.5 m).

##### Heme Reconstitution and Metal Ion Removal

Purified BFR was reconstituted with heme to assure maximal occupancy of heme binding sites (17). Specifically, hemin chloride (Frontier Scientific, Inc.) was dissolved in sodium hydroxide solution (0.1 m), diluted with MES buffer (0.2 m, pH 6.5), and then centrifuged to remove insoluble material. After adding the heme solution to the protein in MES buffer (0.2 m, pH 6.5) containing NaCl (1 m) at 80 °C, the protein-heme solution was incubated at 80 °C (5–10 min) and then cooled to room temperature. Unbound and adventitiously bound heme were removed with an Amicon Ultra 30k centrifugal filter (Millipore) and a PD-10 desalting column (GE Healthcare). Metal ions were removed from the reconstituted protein by heating the protein samples in MES buffer (0.2 m, pH 6.5), EDTA (10 mm), and NaCl (1 m) to 80 °C for 30–60 min before exchanging into MES buffer with Amicon Ultra 30k centrifugal ultrafilters and PD-10 desalting columns.

##### Ferroxidase Kinetics Measurements

Phase 2 of iron oxidation by BFR was observed by monitoring the change in absorbance at 340 nm after the addition of Fe^2+^ to BFR devoid of metal ions with a BioLogic Model SFM-400 stopped-flow spectrometer. Specifically, equal volumes of BFR (1 μm) in MES buffer (0.2 m, pH 6.5) and ferrous ammonium sulfate solution (0, 10, 20, 30, 40, 50, 60, 80, and 100 μm) freshly prepared in HCl (6 mm) were mixed at 25 °C. Phase 3 of iron oxidation by BFR was studied with Cary Models 4000 or 6000i spectrophotometers (Varian). Four additions of ferrous ammonium sulfate solution (200 μm) were made to BFR (0.5 μm) in MES buffer (0.1 m, pH 6.5, 25 °C) at 30-min intervals, and the change in absorbance (340 nm) occurring 20 s after the first iron addition was monitored.

First order rate constants for Phase 2 were calculated from the dependence of the initial rate for the iron oxidation on iron concentration (18). The lag phase exhibited by the D132F variant was not used for the determination of the initial ferroxidase rates. The total change in absorbance after 30 s of Phase 2 reactions was calculated for all variants and taken to be a measure of occupancy of the dinuclear iron site by Fe(III), except for the D132F variant. For this variant, the change in absorbance after 180 s was determined. Replicate measurements were performed, and the associated standard deviations were determined. Phase 3 mineralization rates were calculated as previously described (18).

##### X-ray Crystallographic Analysis

Purified BFR D132F containing <1 heme/24-mer was crystallized by hanging-drop vapor diffusion. Reservoir solutions contained ammonium sulfate (50–80% saturated), NaCl (0.1 m), and Tris HCl (20 mm, pH 7.2) as previously described (19). Crystals were immersed in ammonium sulfate (47% saturated), NaCl (0.1 m), Tris-HCl (20 mm, pH 7.2), and glycerol (30%) for cryoprotection before freezing in liquid nitrogen.

Diffraction data were collected at the Stanford Synchrotron Radiation Lightsource on beamline 9-2, processed with XDS (20), and merged with Aimless (21, 22) from the CCP4 suite (23). The structure was solved by molecular replacement with Phaser (24) using an *E. coli* BFR structure (Protein Data Bank (PDB) ID 2Y3Q (25)) as the search model. The resulting model of 12 complete monomers in the asymmetric unit was refined using non-crystallographic symmetry restraints with Refmac5 (26) and the Phenix suite (27), rebuilt using Coot (28), and validated using MolProbity (29). PyMol (Schrödinger, LLC) was used to generate structural renderings, and APBS (30–32) was used within PyMol to calculate electrostatic potential surfaces with a probe radius of 1.4 Å, protein and external dielectric constants of 2 and 78, respectively, a temperature of 310 K, and an ionic strength of 0.15 m. Structural models for variants not characterized crystallographically were constructed with the program MODELLER (33).

## RESULTS

**FIGURE 2.**
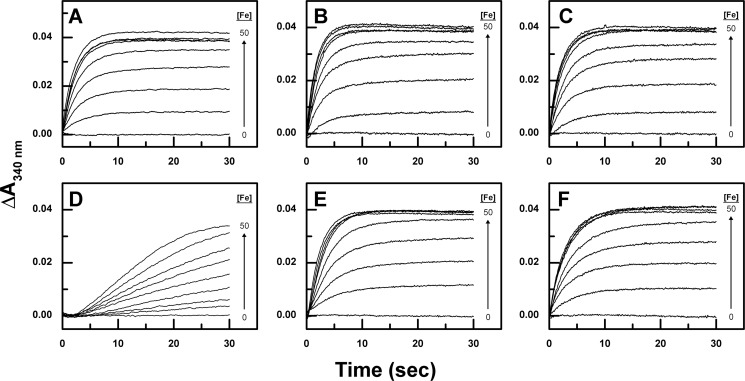
**Phase 2 iron oxidation profiles of wild-type bacterioferritin and B-type variants.** Ferrous ammonium sulfate was added to final concentrations of 0, 5, 10, 15, 20, 25, 30, 40, and 50 μm to 0.5 μm wild-type (*A*), N34F (*B*), E66F (*C*), D132F (*D*), D132N (*E*), and D139F (*F*) BFR in MES buffer (0.1 m, pH 6.5, 25 °C) using a stopped-flow instrument.

Five variants of *E. coli* BFR were constructed in an effort to interfere with possible iron transport through the B-type channel. Three anionic residues, Glu-66, Asp-132, and Asp-139, were selected for substitution based on their location and their negative electrostatic charge. In addition, Asn-34 was selected for substitution because its position allows it to constrict part of the B-type channel. In each case, these residues were replaced with phenylalanine either to remove a negative charge, introduce steric hindrance, or both. In addition, Asp-132 was replaced with asparagine to evaluate the consequences of eliminating a charged group at this position while minimizing any additional stereochemical or hydrogen bonding perturbation. The kinetics of Phase 2 of the ferroxidase activity of all five variants (N34F, E66F, D132F, D132N, and D139F) were studied by stopped-flow spectroscopy (Fig. 2). All but one of the variants included in this analysis exhibited kinetic behavior that was very similar to that of wild-type BFR. The D132F variant was unique in exhibiting significantly decreased reactivity. In addition, this variant also exhibited a lag phase (∼2.5 s) that was not observed for the other variants.

**FIGURE 3.**
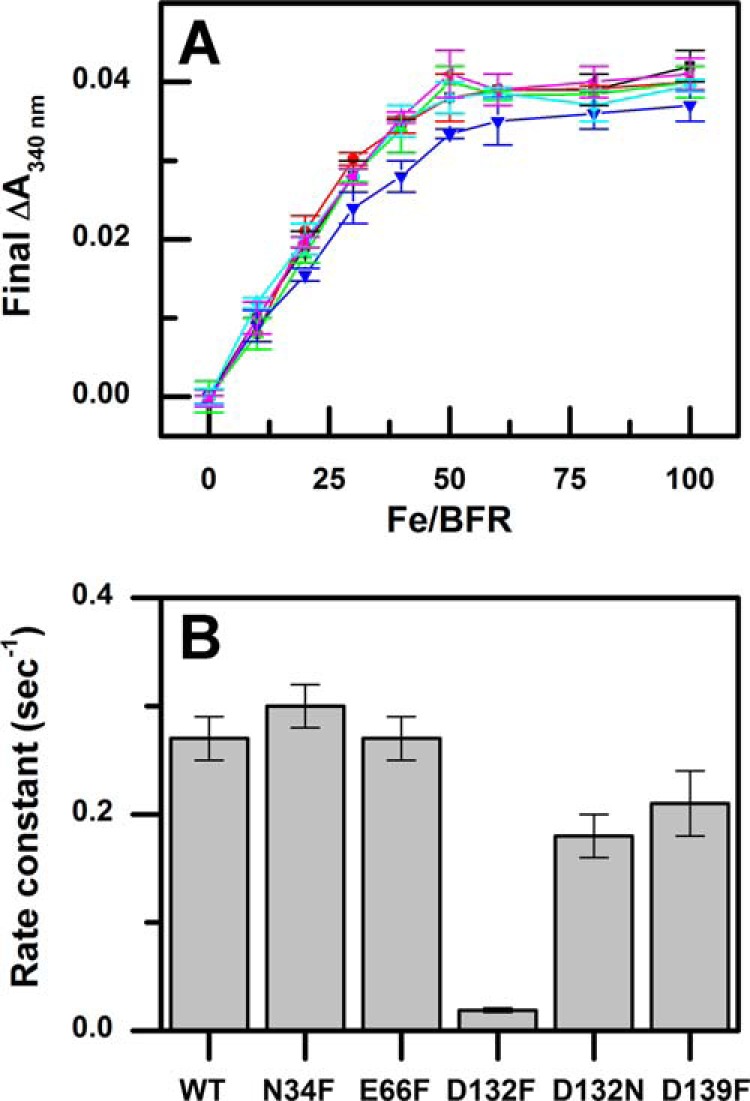
**Phase 2 rate constants and Δ*A*_340 nm_ endpoints for wild-type bacterioferritin and B-type variants.** Endpoints (*A*) and Phase 2 rate constants (*B*) are shown for wild-type (*black square*), N34F (*red circle*), E66F (*green triangle*), D132F (*blue inverted triangle*), D132N (*green diamond*), and D139F (*pink triangle*) BFR.

Previous studies of the kinetics for Phase 2 of the ferroxidase activity of the wild-type protein revealed a plateau in the change of absorbance after the addition of iron in amounts sufficient to fully occupy the dinuclear iron sites (2). Similar behavior was observed for the B-type channel variants in the current study in which saturation was observed after the addition of ∼50 eq of iron for each BFR 24-mer (Fig. 3*A*). The dependence of the initial rates for Phase 2 on Fe^2+^ at non-saturating concentrations exhibits first-order dependence as previously reported (3, 18) and permitted calculation of rate constants for the Phase 2 of the variants (Fig. 3*B*). Although the rate constants obtained for the N34F and E66F variants are similar to that reported for wild-type BFR, the rate constants for the D132N and D139F variants are decreased, and the rate constant for the D132F variant is <110 that of wild-type BFR.

**FIGURE 4.**
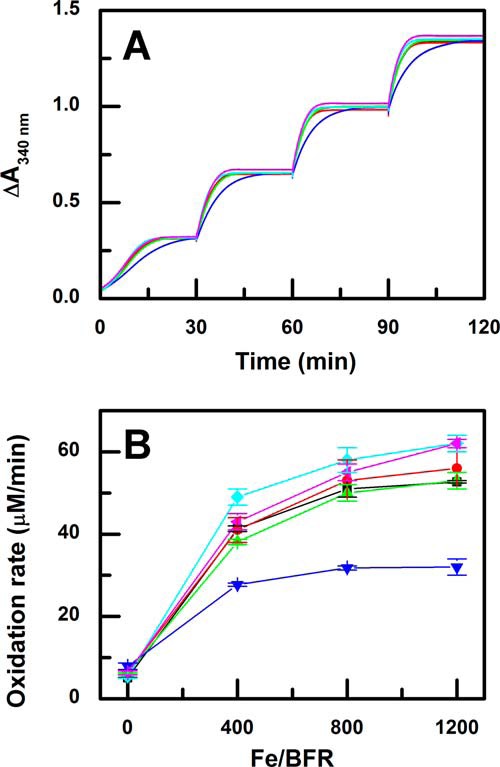
**Phase 3 iron oxidation profiles of wild-type bacterioferritin and B-type variants.** *A*, four additions of 200 μm ferrous ammonium sulfate were made to 0.5 μm wild-type (*black line*), N34F (*red line*), E66F (*lime green line*), D132F (*blue line*), D132N (*green line*), and D139F (*pink line*) BFR in MES buffer (0.1 m, pH 6.5, 25 °C). *B*, initial iron oxidation rates for the four additions in wild-type (*black square*), N34F (*red circle*), E66F (*green triangle*), D132F (*blue inverted triangle*), D132N (*green diamond*), and D139F (*pink triangle*) BFR.

The kinetics of Phase 3 of the ferroxidase reaction, core formation, was monitored after four sequential additions of iron to each of the B-type variants (Fig. 4*A*). The reaction profiles exhibited an expected initial increase in absorbance that corresponds to the Phase 2 reaction. The first addition of iron produced a sigmoidal increase in absorbance that was observed previously for BFR samples with relatively high levels of bound heme (17). As previously reported for the wild-type protein, the rate of iron core formation increases as the iron core size increases (Fig. 4*B*). Relative to the kinetics of Phase 3 observed for wild-type BFR, the reactivities of the N34F and E66F variants are similar, those of the D132N and D139F variants were slightly greater, and that of the D132F variant was clearly diminished.

**FIGURE 5.**
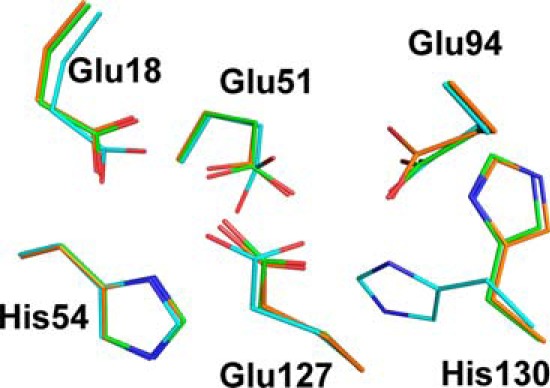
**Superposition of dinuclear iron sites from wild-type bacterioferritin and D132F variant.** Representative dinuclear iron sites of apo-wild-type BFR (PDB IDs 3E1L and 3E1J (4)) with an open His-130 (*green*) and a closed His-130 (*cyan*) were aligned with the D132F variant with an open His-130 (*orange*) for comparison.

The three-dimensional structure of the D132F variant was determined to gain insight into the structural basis for the relatively low reactivity of this variant in both Phase 2 and 3 of the ferroxidase reaction (Table 1), and the coordinates for this structure were deposited in the Protein Data Bank (accession code 4U3G). Crystals of the D132F variant were grown under conditions reported previously for crystallization of the apo form of the wild-type protein (19), and the structure was solved to 2.0 Å resolution by molecular replacement. No major structural differences were observed between the overall structures of the apo-D132F variant and the wild-type protein (PDB ID 3E1L (4)) (root mean square deviation = 0.27 Å for 1896 Cα atoms of 12-mer in the asymmetric unit). Residues forming the dinuclear iron sites that account for the Phase 2 reaction were examined closely to look for any structural changes present in the D132F variant (Fig. 5). The dinuclear iron site is free of metal ions, as expected, and His-130 is located in the “open” position. The dinuclear iron site of the variant aligns closely with sites of the wild-type protein with His-130 in an open position (PDB ID 3E1L (4)) (root mean square deviation = 0.61 Å for 56 atoms) and less well with it in a “closed” position (PDB ID 3E1L (4)) (root mean square deviation = 1.22 Å for 56 atoms). The structural alignments establish that the metal-free dinuclear iron sites of the D132F variant are not significantly different from those in the wild-type structure except for the position of His-130, which is known to adopt alternate conformations.

**TABLE 1:** **Crystallographic data collection and refinement statistics** Statistics for the highest-resolution shell are shown in parentheses. r.m.s.d., root mean square deviation.

Resolution range (Å)	49.15-2.00 (2.07-2.00)
Space group	*P*4_2_2_1_2
Unit cell (Å)	*a* = 208.5, *b* = 208.5, *c* = 143.0
Unique reflections	209745 (20667)
Completeness (%)	99.6 (99.1)
Multiplicity	4.9 (3.7)
Average *I*/σ*I*	11.4 (2.3)
*R*_merge_	0.110 (0.589)
Wilson B-factor (Å^2^)	11.0

**Refinement**	
*R*_work_	0.190 (0.289)
*R*_free_	0.220 (0.324)
No. of atoms	19,281
Protein	15,864
Water	3,337
Sulfate	80
Average B-factor (Å^2^)	15.8
Protein	12.7
Water	30.1
Sulfate	38.7
r.m.s.d.	
Bond lengths (Å)	0.007
Bond angles (°)	0.90
Ramachandran plot (%)	
In favored	100%
In disallowed	0%
PDB accession code	4U3G

**FIGURE 6.**
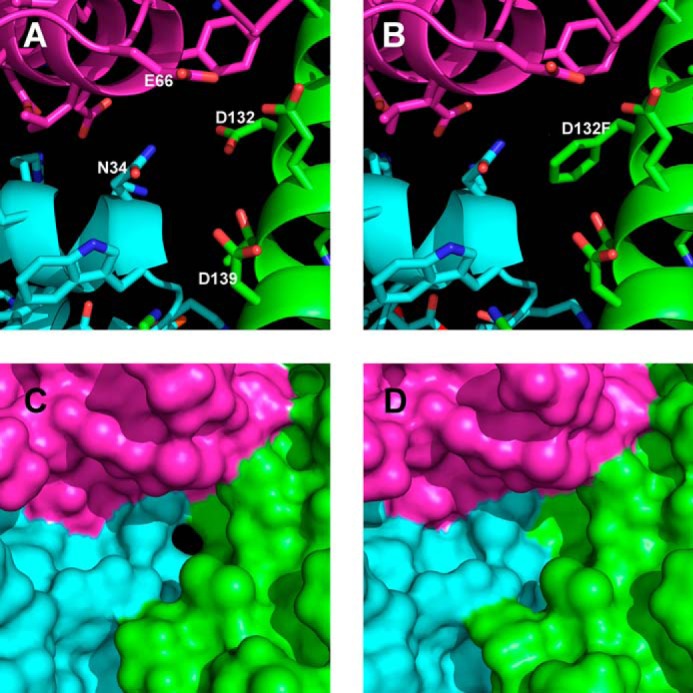
**Comparison of B-type channels in wild-type bacterioferritin and D132F variant.** One of the B-type channels formed at the interface between three subunits is displayed with the separate subunits colored *magenta*, *cyan*, and *green*. The amino acids forming the B-type channels and the molecular surfaces generated with a 0.8 Å solvent probe radius after adding hydrogens in the riding positions are displayed for the wild-type (*A* and *C*; PDB ID 3E1L (4)) and the D132F variant (*B* and *D*).

Comparison of the B-type channels of wild-type BFR and the D132F variant highlighted an important consequence of the mutation (Fig. 6). Calculation of molecular surfaces for wild-type BFR and the D132F variant with the assumption of a solvent probe radius of 0.8 Å allows visualization of channels that would be sufficiently large for passage of Fe(II) ions (ionic radius ∼0.76 Å). Using this probe radius, it can be seen that although the B-type channels in the wild-type structure could provide a passage for iron from bulk solvent to the inner core, replacement of Asp-132 with phenylalanine sterically impedes access via this channel, consistent with the slower rate of iron core growth observed for the Phase 3 kinetics of the variant.

## DISCUSSION

Based on the hypothesis that the B-type channel is an important route of iron entry to the cavity of BFR, various residues surrounding the B-type channel were substituted in an attempt to disrupt normal iron influx. Replacement of B-type channel residues with phenylalanine identified the D132F variant as having significantly decreased Phase 2 and 3 iron oxidation activities (Fig. 3*B* and 4*B*). Although the Phase 2 iron oxidation rate of the D132F variant is significantly decreased, the iron-to-protein stoichiometry of the Phase 2 reaction of this variant is similar to that of the wild-type protein and the other variants (Fig. 3*A*). Thus, the D132F substitution in no way compromises iron binding to the dinuclear site. The structure of the D132F variant confirms that the D132F substitution does not significantly change the overall structure of BFR or, most importantly, that of the dinuclear site where Phase 2 iron oxidation occurs (Fig. 5).

The structure of the D132F variant also demonstrates that this substitution sterically blocks the B-type channel (Fig. 6). The position of residue 132 is critical for the blockage of the B-type channel in the D132F variant because the side chain is located at the constricting point of the channel (Fig. 6*B*). Steric hindrance introduced by the N34F, E66F, and D139F substitutions at the B-type channel was incapable of effectively blocking iron entry as demonstrated by the small changes in iron oxidation rates exhibited by these variants relative to those of wild-type BFR (Figs. 3*B* and 4*B*).

**FIGURE 7.**
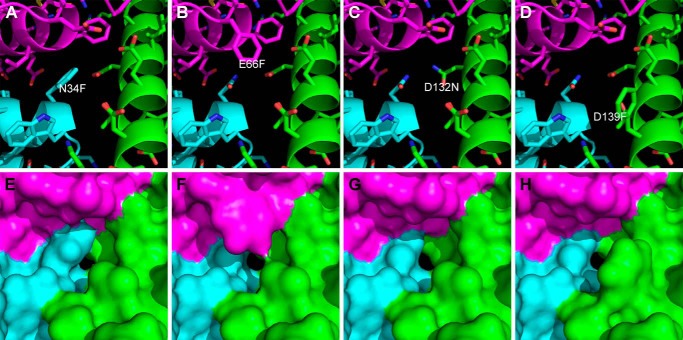
**Comparison of B-type channels in bacterioferritin variants.** Mutations were simulated with the program MODELLER starting with the structure of wild-type bacterioferritin (PDB ID 3E1L). The B-type channels formed at the interface between three subunits are displayed with the individual subunits colored *magenta*, *cyan*, and *green*. The amino acids forming the B-type channels and the molecular surfaces generated with a 0.8 Å solvent probe radius after adding hydrogens in the riding positions are displayed for the N34F (*A* and *E*), E66F (*B* and *F*), D132N (*C* and *G*), and D139F (*D* and *H*) variants.

Consistent with this conclusion, simulated structural models of these variants indicate that these substitutions do not completely obstruct the B-type channel (Fig. 7). Substitution of Asp-132 with asparagine removes a negative charge without introducing steric hindrance and is insufficient to decrease iron oxidation rates substantially. This result confirms that the large decrease in iron oxidation activity exhibited by the D132F variant results from steric hindrance of the channel and not from elimination of the negative charge of the aspartyl residue. Substitution of Glu-66 or Asp-139 with phenylalanine removes negative charges and was also insufficient to decrease iron oxidation rates or to influence transfer of iron through the protein shell significantly.

Our results show that the B-type channels are the major if not the only entry points by which iron accesses the central cavity of *E. coli* BFR. Although iron oxidation by the D132F variant is significantly compromised, abolition of iron oxidation activity was not achieved. This residual activity may result from side chain motion at the channel opening that results in incomplete steric obstruction of the B-type channel. Alternatively, the 3- and 4-fold symmetry channels may account for the residual iron oxidizing activity of this variant.

**FIGURE 8.**
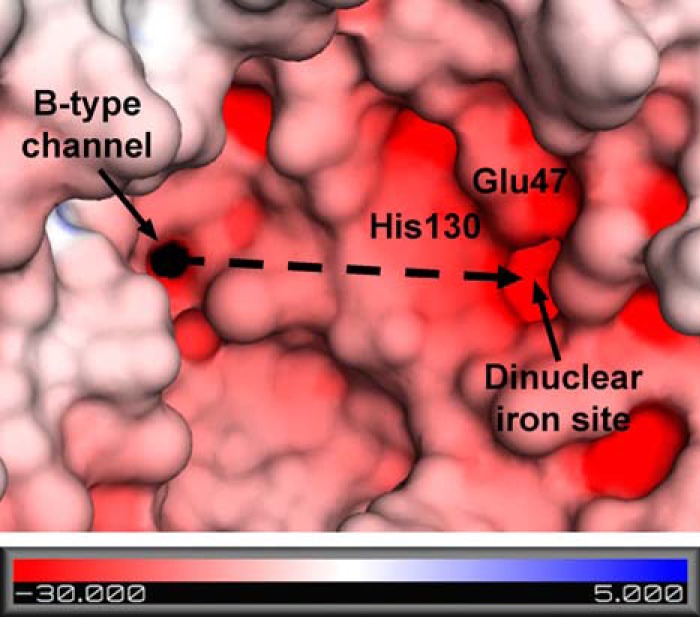
**Predicted path of iron migration from the B-type channel to the dinuclear iron site.** The molecular surface generated with a 1 Å solvent probe radius is colored by the electrostatic potential on the solvent-accessible surface for the wild-type BFR structure in which His-130 is in the open conformation (PDB ID 3E1L (4)). The structure is viewed from the inner core surface to show the B-type channel and the cavity leading to the dinuclear iron site. After iron enters through the B-type channel, it is postulated to travel ∼22 Å toward the dinuclear iron site along a trajectory indicated by the *dashed arrow*.

An important conclusion from the decrease in the Phase 2 iron oxidation rate for the D132F variant is that a significant amount of iron normally enters the central cavity of BFR before entering the dinuclear site where the Phase 2 reaction occurs. This involvement is demonstrated by the reduction in the rate of Phase 2 oxidation for the D132N, D132F, and D139F variants (Figs. 2 and 3). Electrostatic surface potential calculations identify an inner surface patch with negative potential that connects the B-type channel to the dinuclear site (Fig. 8). This surface may provide a route (∼22 Å from the B-type channel to the dinuclear iron site) by which iron is directed toward the dinuclear site and the nucleation site after entering the BFR cavity through the B-type channel. Previous studies of subunit dimer variants of BFR revealed that the E47N substitution significantly lowered the rate of Phase 2 oxidation, demonstrating that this residue helps guide iron to the dinuclear center (5). The present data are consistent with this result because Glu-47 forms part of the route from the B-channel to the dinuclear center.

Thus, binding of Fe(II) at the dinuclear site does not, or at least does not exclusively, occur via the previously proposed ferroxidase pore (4, 7), a relatively hydrophobic route by which dioxygen might access the dinuclear site. For iron to enter the dinuclear site from the cavity side, His-130 would presumably be in the open conformation so that the dinuclear iron site is accessible from the inner cavity surface. Upon binding of two Fe(II) ions at the dinuclear site, His-130 probably switches to the closed conformation before oxidation (4).

**FIGURE 9.**
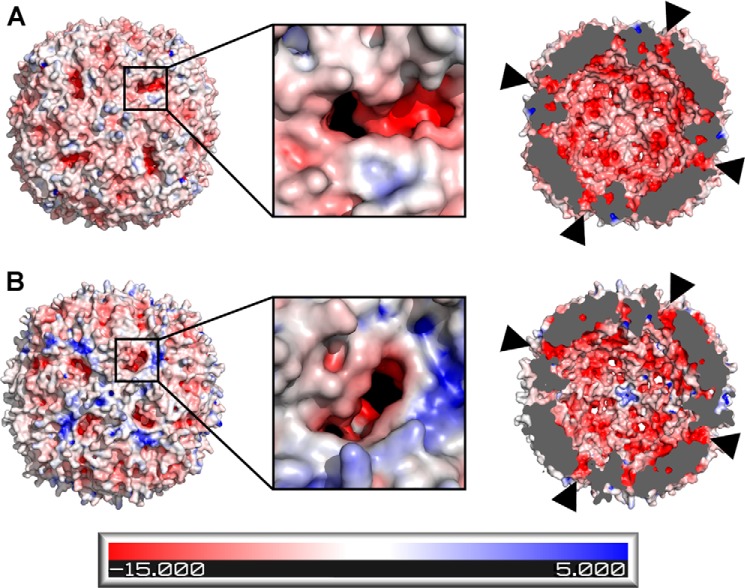
**Electrostatic potential surfaces of ferritin from *E. coli* and *P. furiosus*.** The outer surface, close-up of a B-type channel, and the inner surface of *E. coli* ferritin A (*A*; PDB ID 1EUM) and *P. furiosus* ferritin (*B*; PDB ID 2JD6) are shown. The electrostatic potentials were calculated with APBS running in PyMol. The proteins are oriented so that the 4-fold channel is in the center. B-type channels in the cross-sectional views are indicated with *arrowheads*.

The 3-fold symmetry channels have been shown to provide access of Fe(II) to the central cavity in animal ferritins, and both the 3-fold and 4-fold symmetry channels contribute to iron uptake by plant ferritins (34, 35), but the route of iron passage into neither bacterial nor archaeal ferritins has been established previously. Our experiments with BFR combined with amino acid sequence data provide clear evidence that the B-type channels are important routes of iron uptake by many of these latter forms of ferritin. Examination of ferritins from *E. coli* (36), *Campylobacter jejuni* (PDB ID 1KRQ), *Mycobacterium tuberculosis* (38), *Vibrio cholera* (PBD ID 3QZ3), *Thermotoga maritima* (PDB ID 1Z4A) (40), and *Pyrococcus furiosus* (41) reveals that B-type channels in these structures are larger than those observed in the structure of *E. coli* bacterioferritin and should readily permit iron passage (Fig. 9). Nevertheless, in some cases, B-type channels might not be so involved. For example, in *Pseudomonas aeruginosa* ferritin, the B-type channels are obstructed by a tyrosyl residue (39), whereas in *Helicobacter pylori* ferritin the observation of iron bound to histidyl residues in the 4-fold symmetry channels suggests that this channel may play a role in iron transport (37). Therefore, although the B-type channels may represent a major pathway for iron uptake by many prokaryotic ferritins, structural variations among prokaryotic ferritins may allow other channels to fulfill the same function in other prokaryotic members of this protein family. However, the generally conservative nature of the B-channels in bacterioferritins is consistent with their having a common role of iron entry in most BFRs.

## Footnotes

2 The abbreviation used is:

BFR: bacterioferritin.
